# Whole-exome sequencing identifies novel mutations in ABC transporter genes associated with intrahepatic cholestasis of pregnancy disease: a case-control study

**DOI:** 10.1186/s12884-021-03595-x

**Published:** 2021-02-05

**Authors:** Xianxian Liu, Hua Lai, Siming Xin, Zengming Li, Xiaoming Zeng, Liju Nie, Zhengyi Liang, Meiling Wu, Jiusheng Zheng, Yang Zou

**Affiliations:** 1 Key Laboratory of Women’s Reproductive Health of Jiangxi Province, Jiangxi Provincial Maternal and Child Health Hospital, Nanchang, 330006 Jiangxi China; 2 Central Lab, Jiangxi Provincial Maternal and Child Health Hospital, Nanchang, 330006 Jiangxi China; 3 Department of Obstetrics, Jiangxi Provincial Maternal and Child Health Hospital, Nanchang, 330006 Jiangxi China

**Keywords:** ICP, WES, TBA, ABC transporter genes, Genetic variants, *ABCC2* gene, Ser1342Tyr mutation, Gene expression

## Abstract

**Background:**

Intrahepatic cholestasis of pregnancy (ICP) can cause premature delivery and stillbirth. Previous studies have reported that mutations in ABC transporter genes strongly influence the transport of bile salts. However, to date, their effects are still largely elusive.

**Methods:**

A whole-exome sequencing (WES) approach was used to detect novel variants. Rare novel exonic variants (minor allele frequencies: MAF < 1%) were analyzed. Three web-available tools, namely, SIFT, Mutation Taster and FATHMM, were used to predict protein damage. Protein structure modeling and comparisons between reference and modified protein structures were performed by SWISS-MODEL and Chimera 1.14rc, respectively.

**Results:**

We detected a total of 2953 mutations in 44 ABC family transporter genes. When the MAF of loci was controlled in all databases at less than 0.01, 320 mutations were reserved for further analysis. Among these mutations, 42 were novel. We classified these loci into four groups (the damaging, probably damaging, possibly damaging, and neutral groups) according to the prediction results, of which 7 novel possible pathogenic mutations were identified that were located in known functional genes, including *ABCB4* (Trp708Ter, Gly527Glu and Lys386Glu), *ABCB11* (Gln1194Ter, Gln605Pro and Leu589Met) and *ABCC2* (Ser1342Tyr), in the damaging group. New mutations in the first two genes were reported in our recent article. In addition, compared to the wild-type protein structure, the *ABCC2* Ser1342Tyr-modified protein structure showed a slight change in the chemical bond lengths of ATP ligand-binding amino acid side chains. In placental tissue, the expression level of the *ABCC2* gene in patients with ICP was significantly higher (*P* < 0.05) than that in healthy pregnant women. In particular, the patients with two mutations in ABC family genes had higher average values of total bile acids (TBA), aspartate transaminase (AST), direct bilirubin (DBIL), total cholesterol (CHOL), triglycerides (TG) and high-density lipoprotein (HDL) than the patients who had one mutation, no mutation in ABC genes and local controls.

**Conclusions:**

Our present study provide new insight into the genetic architecture of ICP and will benefit the final identification of the underlying mutations.

**Supplementary Information:**

The online version contains supplementary material available at 10.1186/s12884-021-03595-x.

## Background

Intrahepatic cholestasis of pregnancy (ICP) is a reversible pregnancy-specific liver disease characterized by pruritus and abnormal liver function, such as elevated liver enzymes and increased serum total bile acids (TBA) (≥ 10 μmol/L), that appears in the second and third trimesters of pregnancy and resolves completely after delivery in the early postpartum period [1]. The incidence of ICP disease is reported to be between 0.1 and 15.6% depending on geographical differences [1, 2]. ICP has been associated with adverse fetal outcomes, including spontaneous preterm birth, respiratory distress, low Apgar scores, fetal distress and fetal death [3–7]. For example, Li Li et al. reported that birth size was reduced, whereas the rate of small-for-gestational-age infants was increased across increasing categories of serum total TBA [8]. It has been noted that approximately 2–4% of ICP pregnancies are affected by fetal mortality [9, 10]. The level of TBA increases the risk of fetal morbidity and stillbirth [11–13]. Therefore, untangling the mechanisms of ICP and its association with fetal complications is very important.

The abnormal synthesis, metabolism transport, secretion and excretion of bile acids may lead to ICP disease [14]. Therefore, the etiology contributing to the development of ICP disease is complex and depends on multiple factors, including hormonal, genetic and environmental backgrounds [15]. Familial clustering analysis in pedigree studies showed a high incidence in mothers and sisters of patients with ICP, which might indicate a genetic predisposition for the condition [16–19]. Among these, gene mutations in the hepatocellular transporters of bile salts play a pivotal role in the pathogenesis of ICP [20].

Bile salt transport is the key physiological function of ATP-binding cassette (ABC) membrane proteins, covering seven distinct members: ABCA, ABCB, ABCC, ABCD, ABCE, ABCF and ABCG. Of these genes, *ABCB4*, *ABCB11* and *ABCC2* are functionally known genes that affect the development of ICP [21–23]. Except for the *ABCB4*, *ABCB11*, *ABCC2, ATP8B1*, *TJP2* and *ANO8* genes [23, 24], the role of other ABC transporter genes seems to be less studied. By taking advantage of the high-throughput genotyping technologies in a larger-scale population, the WES approach that combined genotype data for all patients has proven to be far more efficient in anchoring exonic mutations for the target gene at once. In particular, the method can greatly accelerate screening for new potential pathogenicity sites of all mutations. Therefore, examining exonic variants across ICP disease groups likely augments the excavation of novel loci. However, to the best of our knowledge, there have been no reports of the use of WES to identify the genetic variants in the ABC gene series of bile acid transporters for ICP disease.

ICP is a complex disease that is influenced by multiple genes. Previously, we reported the identification of the novel gene *ANO8* as a genetic risk factor for intrahepatic cholestasis of pregnancy in 151 ICP samples [24]. In the present study, we hypothesized that genetic variation in ABC transporters confers susceptibility to ICP. Therefore, we performed WES to extensively investigate the presence of mutations, especially for the discovery of new functional variants, of the ABC gene series involved in bile acid transport in 151 patients with ICP disease and related them to the clinical data and pregnancy outcomes.

## Methods

### Patients and clinical data

A total of 151 pregnant women, who were also used for our previous study [24], were included who did not have other liver diseases and were diagnosed as ICP on the basis of skin pruritus in combination with abnormal liver biochemistry indexes, e.g., TBA, ALT and AST. Among them, the cut-off level of TBA was 10 μmol/L. In addition, 26 clinical features covering six main indicators of maternal and neonatal data, including basic patient features (age, gestational age and body mass index: BMI), ion concentrations (K, Na, Cl, Ca, Mg, and P), routine blood tests (white blood cell count: WBC, red blood cell count: RBC, platelet count: PLT, and red blood cell distribution width.SD: RDW.SD), liver function indexes (TBA, ALT, AST, total bilirubin: TBIL, DBIL, and indirect bilirubin: IDBIL), lipid indexes (CHOL, TG, HDL, low-density lipoprotein: LDL, and uric acid) and outcomes of pregnant women and newborns (birth weight and bleeding amount) were recorded. The concrete methods for these biochemical variables can be found in our previous study [24]. Briefly, the ion concentration, liver function and lipid indexes were determined by an AU5800 automatic biochemical analyzer (Beckman Coulter). Routine blood tests were determined by a Sysmex-xn-2000 automatic blood cell analyzer. We collected all the samples from June 2018 through July 2020. Blood samples were collected from 17 to 41 + 3 weeks of gestation. The delivery gestational age of pregnant women ranged from 28 to 41 + 3 weeks (median: 38 weeks). In addition, we also recruited 1029 pregnant women without ICP disease as negative controls in the same periods, which were also used in our previous study. The controls were in the same gestational age range as the patients with ICP.

### Whole-exome sequencing

A total of 151 genomic DNA samples were extracted from peripheral blood by an Axy Prep Blood Genomic DNA Mini prep Kit (Item No. 05119KC3). To fulfill the requirements for genotyping, the concentration and integrity of all the extracted DNA samples were determined by a Nanodrop-1000 spectrophotometer (Thermo Fisher, USA) and gel electrophoresis, respectively. A total of 151 ICP patient samples were subjected to exome capture with a BGI Exon Kit according to the manufacturer’s protocols. DNA libraries were constructed using the combinatorial probe anchor ligation (cPAL) method. Each resulting qualified captured library was then loaded on BGISEQ-500 sequencing platforms. In addition, whole-exome sequencing was also performed in 1029 controls.

### Variant annotations, filtering and prioritizing

The bioinformatics analysis began with the sequencing data. We carried out the analysis mainly according to Huusko et al. [25]. Briefly, first, joint contamination and low-quality variants (read depth < 15 and genotype quality score < 20) were removed. The reads were mapped to the reference human genome (UCSC GRCh37/hg19) using BWA (Burrows-Wheeler Aligner) software [26]. After that, variant calls were conducted by GATK (v3.7) [27]. Finally, the ANNOVAR tool was applied to perform a series of annotations [28], such as the frequency in the databases, conservative prediction and pathogenicity prediction with available website tools for variants.

**Fig. 1:**
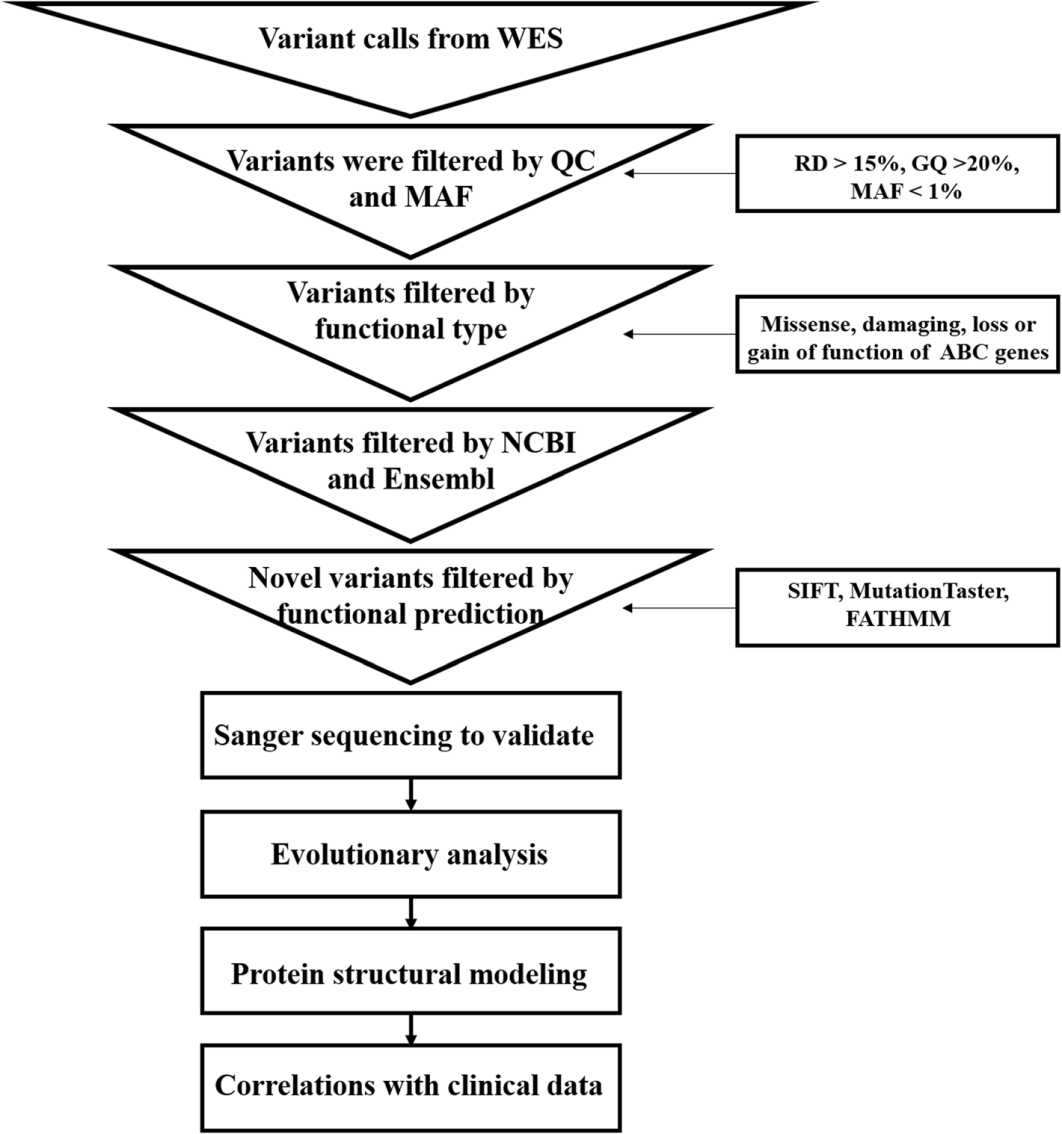
Overview flow chart of the whole-exome sequencing study for intrahepatic cholestasis of pregnancy

The quality control workflow was carried out as shown in Fig. 1. First, we removed variants with an MAF greater than 0.01 in the 1000 Genomes Project (http://www.internationalgenome.org/), Exome Aggregation Consortium (ExAC) (http://exac.broadinstitute.org/), and dbSNP (https://www.ncbi.nlm.nih.gov/snp/) databases. Second, missense, damaging or loss-of-function variants of ABC genes were included in the subsequent analyses. Third, we concentrated on novel variants that were filtered by NCBI and Ensembl. In addition, we prioritized paying attention to novel variants that would likely have functional effects. For instance, a variant was highlighted when it was predicted to be simultaneously deleterious by SIFT, Mutation Taster and FATHMM software.

### Statistical analysis

We applied the *sapply* function to calculate descriptive statistics for clinical data. The Pearson correlation coefficient estimation between clinical data was evaluated by using the *cor* function [29]. The *cor.test* function was used to examine the significance of the correlation coefficients. *Fisher’s* test was used to test the significance of the frequency between 151 patients with ICP and 1029 controls in 3 databases [30]. Moreover, the *pie* function was used to draw the percentage of all ABC mutations. The comparison of average value differences in six clinical datasets (TBA, AST, DBIL, CHOL, TG and HDL) among the four groups was performed by one-way ANOVA [31]. All the analyses were preformed using R software. In addition, we used three web-available tools, namely, SIFT, Mutation Taster and FATHMM, to predict protein damage. Predictions were defined as damaging, probably damaging, possibly damaging, or neutral when all three prediction tools, two out of three, one out of three and none reached the damaging level, respectively.

### Verification of novel candidate sites by sanger sequencing

Since the WES approach is associated with false positive results, the presence of 13 interesting novel mutations from WES analyses was confirmed using Sanger sequencing. The thirteen loci were selected for sequencing mainly based on functional prediction results for mutations (the damaging, probably damaging, possibly damaging, and neutral groups) and classification of members of the ABC transporter gene superfamily (ABCA, ABCB, ABCC, ABCD, ABCE, ABCF and ABCG). The primers were designed by Primer Premier 5 software. The details of the PCR primers and their optimum annealing temperatures are shown in Table 1.

**Table 1:** Primes used to confirm the variants in ABC transporter genes

Order	Gene	Patient	Annealing temperature (°C)	Amplicon (bp)	Forward primer (5′-3′)	Reverse primer (5′-3′)
1	*ABCA2*	ICP64	56	229	CTACCCAGGTCCATCCTGAC	CCACCCACTTGTCCATCTCT
2	*ABCA10*	ICP35	56	158	AACCTATGCTCGCTGGTTTG	TGTTCTTACCTGGGCCATTC
3	*ABCA12*	ICP75	56	150	CATGGATCCGAAGTCGAAA	ACCTTTTCCCACCTGTCATC
4	*ABCA13*	ICP112	56	245	CAAGGACCAAGCATCATTCC	GGGAGGTGGAAGCAGAAGAT
5	*ABCB4*	ICP133, ICP135	50	200	ACATTCCAGGTCCTATTT	TCGAGAAGGGTAAGAAAA
6	*ABCB4*	ICP154	56	463	CTGTCAAAGAGGCCAACG	GCTTCTGCCCACCACTCA
7	*ABCB5*	ICP47	56	460	TCTTACACTAGCCATCCT	CTATTTCCACGACTTACA
8	*ABCB11*	ICP2	56	205	GCTATCGCCAGAGCCCTCA	TACTCCCATCCCTCCCACC
9	*ABCC2*	ICP79	56	368	CCTCTTACCTCCTGTGAC	GTAGACCGTGGAATTGAC
10	*ABCC3*	ICP93	56	189	CCCCAAACTCCTTCTCCC	TGGCATCCACCTTAGTATCA
11	*ABCC9*	ICP124	56	166	TCCCACAACCCATTCATCTT	GCCTCTGCCAACTTTGTAGC
12	*ABCG1*	ICP113	56	212	CTCACGGTGCCTCTTGACTT	GGTGTCATTCAGGCTGACCT
13	*ABCG2*	ICP6	56	180	GTGGCCTTGGCTTGTATGAT	GATGGCAAGGGAACAGAAAA

### Evolutionary conservation analysis

We also selected the same twelve loci as Sanger sequencing for evolutionary conservation analysis for the same reasons. Evolutionary conservation analysis [32, 33] of the amino acids encoded by the new pathogenicity sites in ABC genes was performed among vertebrates, including Gibbon, Macaque, Olive Baboon, Gelada, Marmoset, Mouse, Rat, Cow, Sheep, Pig, Chicken, Zebra Finch, and Zebrafish, using genomic alignments of the Ensembl Genome Browser.

### Protein structure modeling

Protein structure modeling involves in two steps. First, the reference and modified (*ABCC2* Ser1342Tyr) protein sequences were submitted to SWISS-MODEL (http://swissmodel.expasy.org/) software for structure modeling. Then, these two protein models were compared simultaneously using the Chimera 1.14rc package.

## Results

### Whole-exome sequencing results of variants of ABC transporter genes in 151 ICP samples

**Fig. 2:**
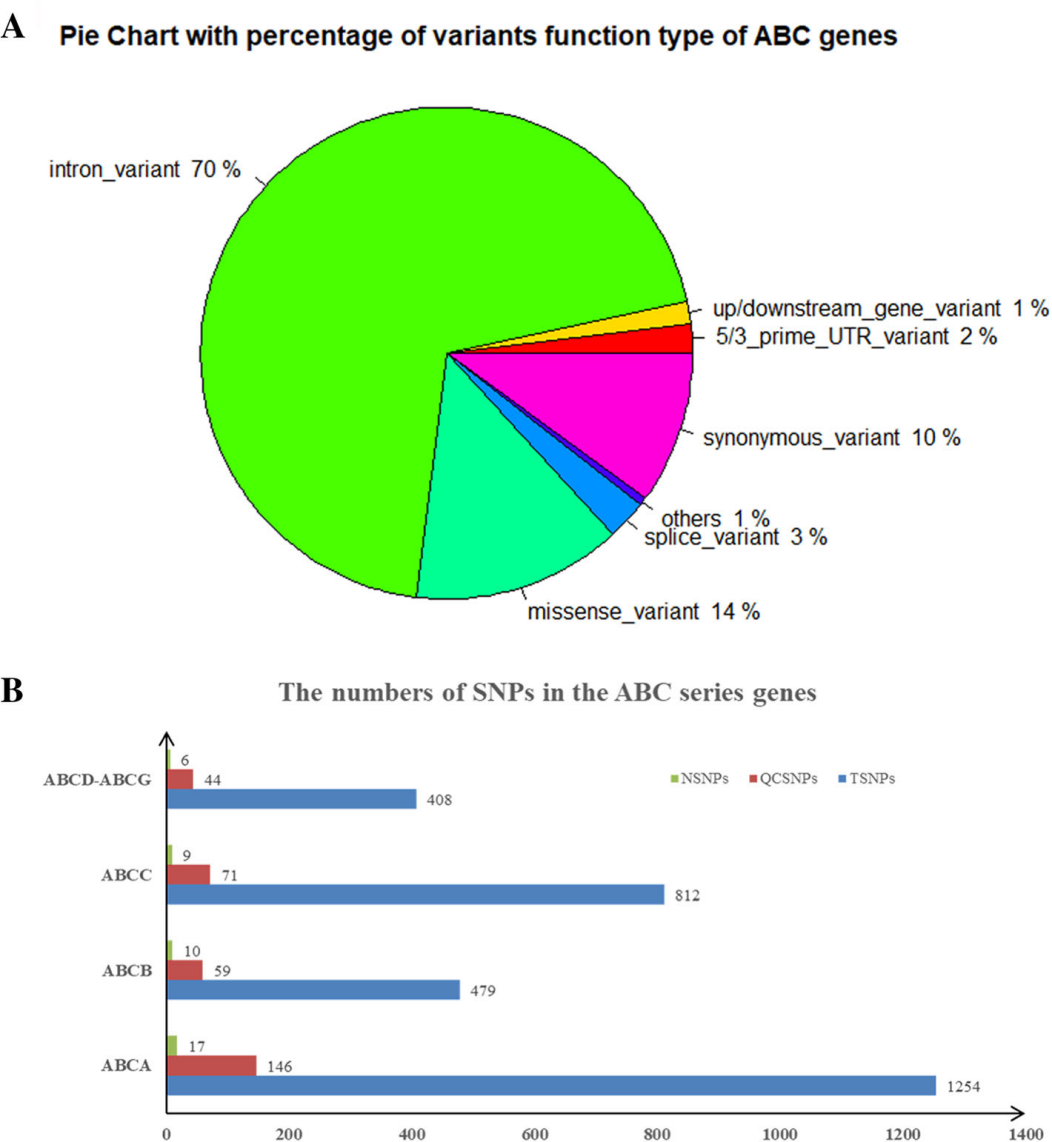
The distribution and numbers of genetic variants from WES data for ICP. **a** The percentage of the types of genetic variants in the ABC series of genes. **b** The total number of genetic variants before and after quality control and the novel variants. TSNPs: Total SNPs before quality control; QCSNPs: Total SNPs after quality control; NSNPs: Novel SNPs

In total, ABC transporters covering 44 genes underwent targeted WES to identify variants in a cohort of 151 patients with ICP disease. Overall, we identified 2953 genetic variants, including 1254 mutations in 12 ABCA genes that consisted of *ABCA1*-*ABCA10* and *ABCA12*-*ABCA13*, 479 variants in *ABCB1* and *ABCB4*-*ABCB11*, 812 genetic variants in eleven genes covering *ABCC1*-*ABCC6* and *ABCC8*-*ABCC12*, and 408 variants in the ABCD-ABCG gene series. These types of variants covered 2057 introns, 297 synonymous, 76 splice, 13 stop gained/start lost, 36 3′ primer UTR, 21 5′ primer UTR, 10 downstream gene, 32 upstream gene, 3 structural interaction and 408 missense. The percentage of these types of variants is shown in Fig. 2a. After quality control (MAF < 0.01 in all databases), 42 out of 320 variants were detected that were first reported, including 17 out of 146 variants in ABCA genes, 10 out of 59 in ABCB, 9 out of 71 in ABCC and 6 out of 44 in ABCD-ABCG genes (Fig. 2b).

For these 42 novel mutations, we classified these mutations as four echelons (damaging, probably damaging, possibly damaging and neutral) according to the prediction results (Table 2). The damage group had fifteen mutations, which contained six mutations in three known functional genes associated with ICP disease, such as *ABCB4* Trp708Ter, Gly527Glu and Lys386Glu and *ABCB11* Gln1194Ter, Gln605Pro and Leu589Met. These six novel mutations have been shown in our recently published article [24]. In addition, 9 functional mutations were found in other ABC genes, i.e., *ABCA4* Phe754Ser, *ABCA12* Cys2440Tyr, *ABCA13* Ser3286Ter, *ABCB1* Pro693Ser, *ABCB5* Ser925Ile, *ABCC2* Ser1342Tyr *ABCC3* Ile1147Thr, *ABCC9* Ala456Thr and *ABCG2* Leu646Met. The frequency of these mutations in 151 ICP samples reached 10.60% (16/151). Moreover, they were absent in 1029 local healthy women. Therefore, there was a significant difference in frequency between the case and control groups (*P* = 1.10E-14).

**Table 2:** Forty-two potential pathogenic novel ABC mutations were identified in 151 Han Chinese patients with ICP disease

ID	Gene	Patients	Chr	Position	Alleles	Protein change	SIFT^d^	Mutation Taster^e^	FATHMM	Comprehensive prediction result^f^	GERP++NR^g^	PhastCons100way_ver^h^	MAF in controls	MAF in 151 ICP	***P*** value (controls-ICP)	MAF in databases
1	*ABCA4*	ICP76	chr1	94,522,278	A/G	Phe754Ser	0.001 (D)	0.999 (D)	−4.24 (D)	Damaging	5.66	1	0/(2*1029)	10.60% (16/151)	1.10E-14	Absent in 1000G/ExAC/dbSNP/ChinaMAP databases
2	*ABCA12*^a,b^	ICP75	chr2	215,809,749	C/T	Cys2440Tyr	0.041 (D)	1 (D)	−3.67 (D)	Damaging	5.49	1				
3	*ABCA13*^a^	ICP112	chr7	48,354,004	C/G	Ser3286Ter	–	1 (A)	–	Damaging	5.68	1				
4	*ABCB1*	ICP121	chr7	87,173,579	G/A	Pro693Ser	0.031 (D)	1 (D)	−2.21 (D)	Damaging	5.91	1				
5	*ABCB4*	ICP21	chr7	87,053,310	C/T	Trp708Ter	–	1 (A)	–	Damaging	6.11	1				
6	*ABCB4*^a,b^	ICP154	chr7	87,069,134	C/T	Gly527Glu	0.0 (D)	1 (D)	−2.35 (D)	Damaging	5.61	0.999				
7	*ABCB4*^a,b^	ICP133,135	chr7	87,073,053	T/C	Lys386Glu	0.002 (D)	1 (D)	−2.65 (D)	Damaging	5.47	0.999				
8	*ABCB5*^a,b^	ICP47	chr7	20,767,985	G/T	Ser925Ile	0.002 (D)	0.786 (D)	−2.5 (D)	Damaging	3.91	0.999				
9	*ABCB11*	ICP115	chr2	169,783,704	G/A	Gln1194Ter	–	1 (A)	–	Damaging	5.72	0.993				
10	*ABCB11*	ICP118	chr2	169,826,057	T/G	Gln605Pro	0.006 (D)	0.972 (D)	−1.99 (D)	Damaging	5.5	0.835				
11	*ABCB11*^a,b^	ICP2	chr2	169,826,599	G/T	Leu589Met	0.0 (D)	0.994 (D)	−2.82 (D)	Damaging	5.2	0.959				
12	*ABCC2*^a,b,c^	ICP79	chr10	101,605,418	C/A	Ser1342Tyr	0.0 (D)	0.999 (D)	−2.95 (D)	Damaging	5.79	1				
13	*ABCC3*^a,b^	ICP93	chr17	48,755,166	T/C	Ile1147Thr	0.0 (D)	1 (D)	−2.57 (D)	Damaging	5.7	1				
14	*ABCC9*^a,b^	ICP124	chr12	22,061,100	C/T	Ala456Thr	0.001 (D)	1 (D)	−2.58 (D)	Damaging	5.29	1				
15	*ABCG2*^a,b^	ICP6	chr4	89,013,418	G/T	Leu646Met	0.01 (D)	0.998 (D)	−2.33 (D)	Damaging	5.55	1				
16	*ABCA2*^a,b^	ICP64	chr9	139,910,497	G/C	Asp1108Glu	0.043 (D)	0.999 (N)	−3.27 (D)	Pro damaging	4.2	1	0/(2*1029)	13.91% (21/151)	2.20E-16	Absent in 1000G/ExAC/dbSNP/ChinaMAP databases
17	*ABCA2*	ICP19	chr9	139,913,419	G/A	Ala583Val	0.351 (T)	0.999 (D)	−2.17 (D)	Pro damaging	4.31	0.94				
18	*ABCA5*	ICP102	chr17	67,266,794	A/G	Val997Ala	0.527 (T)	0.961 (D)	−1.77 (D)	Pro damaging	5.45	1				
19	*ABCA7*	ICP108	chr19	1,045,131	C/A	Pro449His	0.015 (D)	1 (N)	−4.44 (D)	Pro damaging	3.05	0				
20	*ABCA8*	ICP47	chr17	66,938,153	A/G	Val8Ala	0.009 (D)	0.984 (N)	−2.41 (D)	Pro damaging	4.84	0.018				
21	*ABCA10*^a,b^	ICP35	chr17	67,212,035	A/G	Leu260Ser	0.038 (D)	1 (N)	−2.28 (D)	Pro damaging	3.21	0.003				
22	*ABCA12*	ICP95	chr2	215,865,543	A/G	Ile1022Thr	0.093 (T)	0.994 (D)	−3.77 (D)	Pro damaging	5.73	1				
23	*ABCA13*	ICP18	chr7	48,559,709	C/G	Leu4624Val	0.017 (D)	0.954 (N)	−2.477 (D)	Pro damaging	5.35	0.702				
24	*ABCA13*	ICP107	chr7	48,634,399	A/G	Thr4912Ala	0.001 (D)	0.958 (N)	−5.88 (D)	Pro damaging	5.7	1				
25	*ABCB9*	ICP112	chr12	123,430,667	C/T	Glu386Lys	0.891 (T)	0.682 (D)	−2.4 (D)	Pro damaging	5	0.979				
26	*ABCB9*	ICP140	chr12	123,433,209	T/C	Ile339Val	0.176 (T)	0.999 (D)	−2.6 (D)	Pro damaging	5.43	1				
27	*ABCC1*	ICP98	chr16	16,149,964	A/G	Ser497Gly	0.268 (T)	0.972 (D)	−2.71 (D)	Pro damaging	5.32	1				
28	*ABCC3*	ICP57	chr17	48,734,467	C/T	Leu137Phe	0.011 (D)	1 (D)	0.85 (T)	Pro damaging	5.38	1				
29	*ABCC5*	ICP49	chr3	183,670,989	T/G	Gln851Pro	0.026 (D)	0.999 (D)	0.95 (T)	Pro damaging	5.62	1				
30	*ABCC6*	ICP57	chr16	16,259,657	G/T	His1043Gln	0.029 (D)	0.999 (N)	−3.35 (D)	Pro damaging	5.52	1				
31	*ABCC9*	ICP36	chr12	21,968,809	T/A	Glu1304Val	0.111 (T)	0.999 (D)	−2.61 (D)	Pro damaging	4.95	1				
32	*ABCC12*	ICP155	chr16	48,180,227	G/A	Pro37Ser	0.217 (T)	0.812 (D)	−2.76 (D)	Pro damaging	5.45	0.974				
33	*ABCD4*	ICP95	chr14	74,757,137	G/A	Ser395Phe	0.033 (D)	0.983 (N)	−2.77 (D)	Pro damaging	4.84	0.875				
34	*ABCD4*	ICP142	chr14	74,757,168	T/C	Thr385Ala	0.254 (T)	0.990 (D)	−2.63 (D)	Pro damaging	4.84	1				
35	*ABCF2*	ICP84	chr7	150,923,406	C/T	Gly47Ser	0.156 (T)	0.999 (D)	−2.77 (D)	Pro damaging	4.81	1				
36	*ABCG1*^a,b^	ICP113	chr21	43,704,752	A/G	Ile273Val	0.002 (D)	1 (D)	0.52 (T)	Pro damaging	4.22	1				
37	*ABCA3*	ICP22	chr16	2,348,506	T/C	Ser593Gly	0.126 (T)	1 (N)	−3.3 (D)	Pos damaging	6.17	0.005	0/(2*1029)	3.31% (5/151)	3.74E-08	Absent in 1000G/ExAC/dbSNP/ChinaMAP databases
38	*ABCA10*	ICP64	chr17	67,181,648	G/C	Leu823Val	0.09 (T)	1 (N)	−2.19 (D)	Pos damaging	2.92	0.011				
39	*ABCA12*	ICP71	chr2	215,802,301	T/C	Asn2492Ser	0.403 (T)	0.958 (N)	−2.28 (D)	Pos damaging	5.79	0.999				
40	*ABCA13*	ICP111	chr7	48,349,554	G/A	Ser3111Asn	0.071 (T)	0.999 (N)	−2.11 (D)	Pos damaging	5.59	0.354				
41	*ABCG1*	ICP22	chr21	43,708,158	C/T	Thr378Ile	0.21 (T)	1 (N)	−1.98 (D)	Pos damaging	4.17	0				
42	*ABCA13*	ICP52	chr7	48,318,169	A/G	Ile2460Val	0.916 (T)	1 (N)	0.66 (T)	Neutral	4.92	0	0/(2*1029)	0.66% (1/151)	0.13	Absent in 1000G/ExAC/dbSNP/ChinaMAP databases

^a^ These loci were validated by Sanger sequencing

^b^ These loci were identified by evolutionary conservation analysis

^c^ The effect of the change in this mutation on the encoded protein structure

^d^ D: disease-causing, T: tolerated

^e^ N: polymorphism, A: automatically disease causing

^f^ Pro damaging: probably damaging; Pos damaging: possibly damaging

^g^ GERP++NR: GREP++ conservation score. The higher the value is, the more conservative the loci

^h^ PhastCons100way_ver: conservative prediction in 100 vertebrates. The scores of the PhastCons score ranged from 0 to 1, and the higher the values are, the more conservative the loci

Twenty-one mutations were assigned to the second tier (probably damaging), including 9 in ABCA genes (*ABCA2* Asp1108Glu and Ala583Val, *ABCA5* Val997Ala, *ABCA7* Pro449His, *ABCA8* Val8Ala, *ABCA10* Leu260Ser, *ABCA12* Ile1022Thr, *ABCA13* Leu4624Val and Thr4912Ala), Glu386Lys and Ile339Val in all ABCB9 genes, 6 in the ABCC gene series (*ABCC1* Ser497Gly, *ABCC3* Leu137Phe, *ABCC5* Gln851Pro, *ABCC6* His1043Gln, *ABCC9* Glu1034Val and *ABCC12* Pro37Ser), *ABCD4* Ser395Phe and Thr385Ala, *ABCF2* Gly47Ser and *ABCG1* Ile273Val in the ABCD-ABCG gene series. These mutations were also absent in controls. The MAF in these mutations between cases and controls showed a significant difference (*P* = 2.20E-16).

We identified five possible potential pathogenic mutations associated with ICP disease, including *ABCA3* Ser593Gly, *ABCA10* Leu823Val, *ABCA12* Asn2492Ser, *ABCA13* Ser3111Asn and *ABCG1* Thr378Ile. Only one mutation was placed in the neutral group. All 42 novel mutations were absent in the databases, including the 1000 Genome Project, ExAC, dbSNP, ChinaMAP and 1029 local controls.

### Confirmation of the novel variants by sanger sequencing

**Fig. 3:**
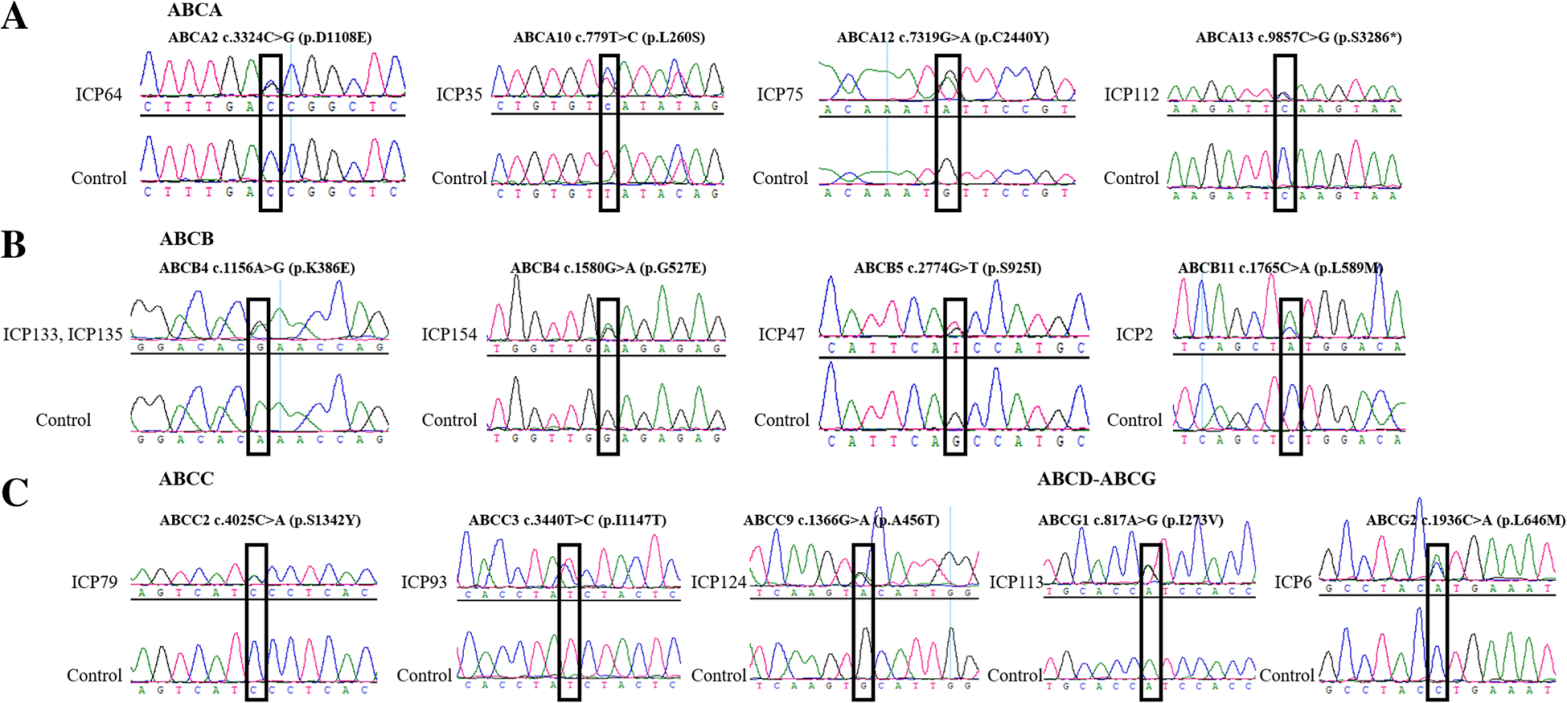
Sanger sequencing to validate the novel variants in the ABC gene series. **a** ABCA genes; **b** ABCB genes; **c** ABCC genes; **d** ABCD-ABCG genes

We used Sanger sequencing to confirm 13 possible candidate novel pathogenicity loci in the ABC family genes from four lines. The results (Fig. 3) were all consistent with WES.

### Evolutionary conservation analysis

**Fig. 4:**
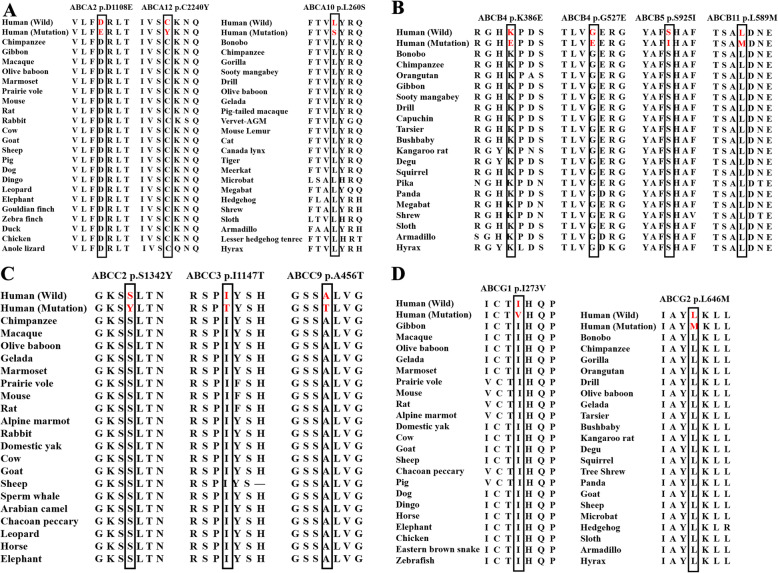
Evolutionary conservation analysis of novel ABC representative mutations. Mutations are highly conserved in vertebrate species

We performed evolutionary conservation analysis of twelve loci, which were also sequenced by Sanger sequencing. The results suggested that these mutations were highly conserved among vertebrate species, including rats, sheep, cows, pigs, dogs, horses, chickens and so on (Fig. 4).

### Comparison of the protein structural model of the *ABCC2* Ser1342Tyr mutation

**Fig. 5:**
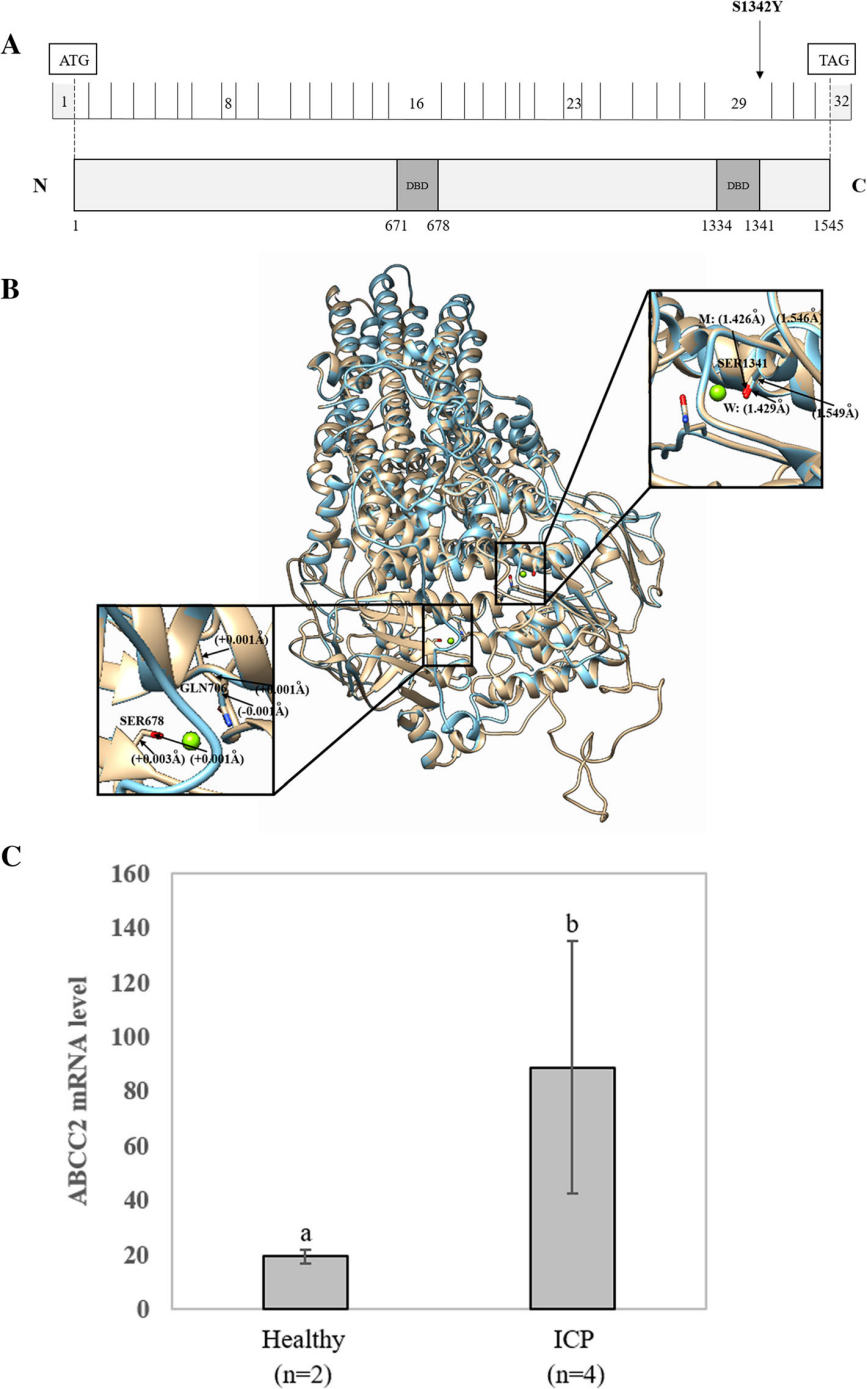
Effects of the *ABCC2* Ser1342Tyr variant on the protein sequence and structure. **a** Distribution of the *ABCC2* variants. *ABCC2* is a 1545-amino acid protein containing two major nucleotide-binding functional domains (ATP binding cassette domains). The locations of two *ABCC2* variants from whole-exome sequencing are shown in the protein sequence. **b** In silico comparison of the higher assembly of the reference and modified (Ser1342Tyr) *ABCC2* protein models. The reference and Ser1342Tyr molecules are presented as gold and blue rounded ribbon structures, respectively. The enlarged image shows that the functional domains have small changes in the chemical bond lengths. DBD: DNA-binding domain. **c** Comparison of the expression level of the *ABCC2* gene between two healthy controls and four patients with ICP. Different letters above the expression column denote significant differences (*P* < 0.05)

*ABCC2* consists of 32 exons involved in bile formation that mediate hepatobiliary excretion of numerous organic anions and conjugated organic anions such as methotrexate and transport sulfated bile salts such as taurolithocholate sulfate [34, 35]. This gene has two main nucleotide binding domains located at positions 671–678 and 1334–1341 (Fig. 5a). We only identified the *ABCC2* Ser1342Tyr mutation when the quality control of the minor allele frequency was below 0.01. The location of this variant (Ser1342Tyr) is close to the ATP binding functional domain (1334–1341). In addition, the mutation was found to be harmful to the function of the protein according to the results of three prediction software programs.

To further investigate the possible effects of the missense variant on protein structure, the reference and modified protein structures of the *ABCC2* gene were compared simultaneously using UCSF Chimera 1.14rc. The results showed that, compared with the reference molecular structure, the 3D model of mutation had a slight change in the chemical bond lengths of ATP-ligand binding amino acid side chains at positions Ser1342, Ser678 and Gln706 (Fig. 5b). The change in the amino side chains could affect the binding efficiency of the ATP molecule.

**Fig. 6:**
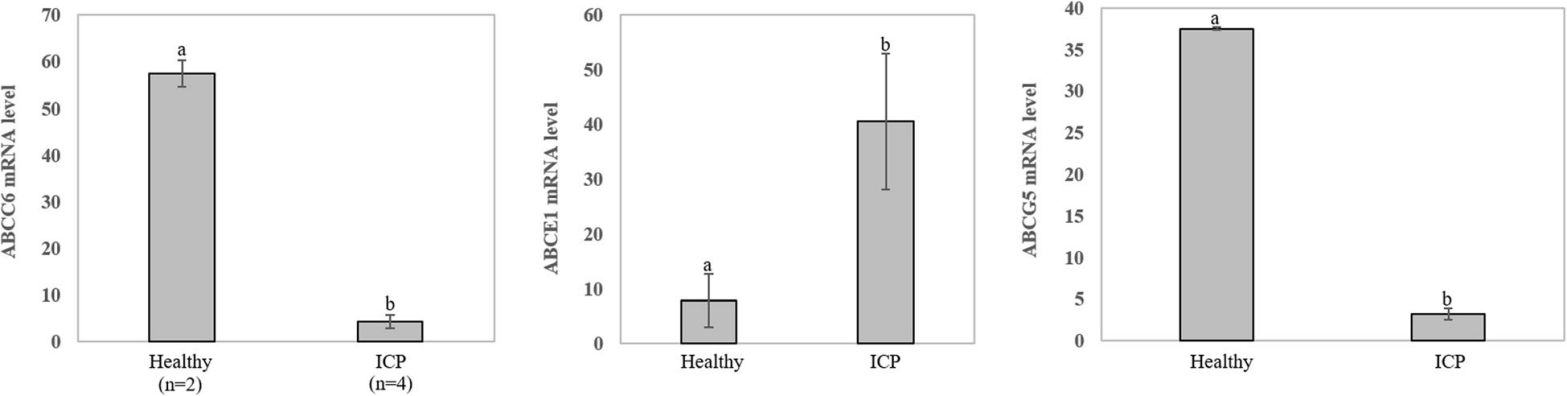
Comparison of the expression levels of the *ABCC6*, *ABCE1* and *ABCG5* genes in placental tissue between two healthy controls and four patients with ICP

In this study, we did not collect placental tissue from these 151 patients with ICP. Therefore, to further analyze the genetic basis of *ABCC2*, we analyzed the mRNA expression level of the *ABCC2* gene in placental tissue between 2 healthy participants and four patients with ICP using GEO datasets derived from NCBI (GEO accession: GSE46157) from Du Q et al.*’s* report [36]. A significant (*P* < 0.05) difference in gene expression was observed between the two groups (Fig. 5c). The expression of *ABCC2* was upregulated in the ICP group. In addition, we also detected that the expression of three other genes, namely, *ABCC6*, *ABCE1* and *ABCG5*, changed in placental tissue (Fig. 6).

### Correlations among clinical data

We examined the correlation coefficients (Supplementary file 1) among these 26 clinical features and found that TBA was significantly negatively correlated with gestation days (r = − 0.34), birth weight (r = − 0.30), and RDW.SD (r = − 0.16) and significantly positively correlated with TBIL (r = 0.52), DBIL (r = 0.56), IDBIL (r = 0.18), ALT (r = 0.17), and AST (r = 0.18). There was also a close relationship among liver function indexes. For example, ALT was highly positively correlated with AST (r = 0.93). ALT and AST were positively correlated with TBIL and DBIL. TBIL positively correlated with DBIL (r = 0.89). CHOL, TG, LDL and uric acid were also positively correlated with ALT and AST. In addition, the concentrations of Ca and Mg ions were positively correlated with AST. The correlations between the abovementioned clinical data were significant (*P* < 0.05).

### Biochemical and clinical features of ICP cases with variants

Descriptive statistics of 26 clinical features for patients with ICP with 42 new mutations are shown in Table 3. For all the clinical data, the levels of ALT, AST, TBA, DBIL, CHOL and TG in 151 patients with ICP disease were higher than the reference levels. Notably, individuals with the novel ABC mutations had a fourfold higher TBA and twofold higher ALT, AST, and TG average values than the reference values, which confirms that ICP disease presents with abnormal liver function and elevated bile acids associated with abnormal lipid metabolism. Compared to the ICP group without the 42 novel mutations, the group with the 42 novel mutations had a higher age, BMI, and levels of K, Na, Cl, RBC, TBIL, IDBIL, TG, and HDL.

**Table 3:** Descriptive statistics of 26 clinical features of ICP individuals associated with/without 42 novel mutations

Features	ICP with 42 novel mutations					ICP without 42 novel mutations					
	N	Mean	SD	Min.	Max.	N	Mean	SD	Min	Max	*P*
**Basic information**											
Age (years)	37	29.54	5.75	17.00	41.00	113	29.35	5.12	20.00	43.00	0.84
Gestational age (days)	34	254.03	22.68	196.00	290.00	110.	260.04	18.05	204.00	290.00	0.06
BMI (kg/m^2^)	31	26.91	3.78	21.50	36.30	106	25.46	3.22	19.60	38.50	0.037
**Ion concentrations**											
K (mmol/L)	34	4.04	0.30	3.60	4.90	106	3.99	0.32	3.20	4.80	0.44
Na (mmol/L)	34	137.71	2.70	133.00	143.00	105	137.36	2.27	132.00	143.00	0.47
Cl (mmol/L)	34	104.26	2.81	97.00	110.00	105	104.08	2.80	98.00	112.00	0.73
Ca (mmol/L)	34	2.31	0.15	2.09	2.80	105	2.31	0.16	2.00	2.90	0.86
Mg (mmol/L)	34	0.80	0.10	0.60	1.10	105	0.82	0.16	0.65	1.89	0.36
P (mmol/L)	34	1.10	0.17	0.72	1.50	105	1.13	0.18	0.70	1.60	0.51
**Routine blood tests**											
WBC (×10^9^)	36	8.21	1.97	4.94	13.36	113	8.66	3.07	4.37	24.23	0.30
RBC (×10^9^)	36	3.89	0.44	3.24	4.98	113	3.82	0.41	2.96	4.81	0.39
PLT (×10^9^)	36	196.33	62.49	87.00	412.00	113	198.81	63.24	75.00	377.00	0.84
RDW.SD (fL)	36	45.23	5.73	36.90	67.30	113	46.08	4.28	36.20	62.40	0.41
**Liver function indexes**											
ALT (U/L)	37	90.81	126.35	3.00	452.00	113	95.77	125.18	4.00	595.00	0.83
AST (U/L)	37	76.35	88.34	12.00	359.00	113	83.08	97.35	12.00	456.00	0.71
TBA (μmol/L)	37	40.58	21.96	12.50	105.80	113	44.73	44.17	10.70	286.80	0.42
TBIL (μmol/L)	36	14.99	8.02	6.90	41.90	112	14.85	7.54	5.70	64.80	0.92
DBIL (μmol/L)	36	6.42	6.06	2.00	28.80	112	6.44	5.98	0.90	49.50	0.99
IDBIL (μmol/L)	36	8.57	4.41	3.50	26.90	112	8.43	3.30	2.90	23.40	0.86
**Lipid index**											
CHOL (mmol/L)	35	6.35	1.66	3.35	10.50	108	6.40	1.43	3.75	10.95	0.87
TG (mmol/L)	35	3.78	1.65	2.11	10.44	108	3.54	1.54	1.20	11.10	0.44
HDL (mmol/L)	35	1.95	0.54	1.28	4.06	108	1.89	0.40	0.92	3.19	0.59
LDL (mmol/L)	35	2.68	1.41	0.19	5.96	108	2.89	1.21	0.13	6.28	0.39
Uric acid (μmol/L)	34	313.74	91.93	131.00	574.00	106	322.41	78.30	111.00	498.00	0.59
**Outcomes of pregnant women and newborns**											
Birth weight (kg)	28	3.04	0.71	1.48	5.30	91	3.04	0.57	1.23	4.10	0.97
Bleeding amount (ml)	28	240.36	62.63	150.00	400.00	86	258.84	102.10	90.00	810.00	0.27

*BMI* body mass index, *WBC* white blood cell, *RBC* red blood cell, *PLT* platelet, *RDW.SD* red blood cell distribution width.SD, *ALT* alanine transaminase, *AST* aspartate transaminase, *TBA* total bile acids, *TBIL* total bilirubin, *DBIL* direct bilirubin, *IDBIL* indirect bilirubin, *CHOL* total cholesterol, *TG* triglyceride, *HDL* high-density lipoprotein, *LDL* low-density lipoprotein

**Fig. 7:**
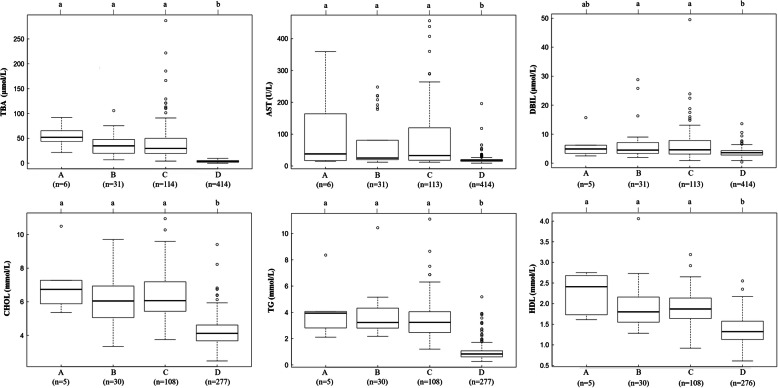
Effects of ABC gene mutations on biochemical indexes. The difference in the average TBA, AST, DBIL, CHOL, TG and HDL in the following four groups: **a** having both mutations, **b** one mutation, **c** no mutation in ABC series genes with ICP, and **d** healthy controls without ICP disease. Means of different groups in rows with different superscript letters are significantly (*P* < 0.001) different from each other

Additionally, we found that six patients with ICP containing two mutations exhibited higher TBA, AST, DBIL, CHOL, TG and HDL than 31 patients with one mutation, 114 patients with ICP with no mutations and 414 local healthy controls (Fig. 7). In particular, for TBA, as a clinical characteristic of ICP, the trends for the average values measured in patients with ICP with mutations of ABC transporter genes and local healthy controls were ranked as follows: ICP with two mutations > ICP with one mutation > ICP with no mutations > healthy controls.

## Discussion

This study is the first to use whole-exome sequencing technology to uncover potential novel pathogenic mutations in ABC family genes involved in bile acid transport. Altogether, we identified 42 new loci covering 44 ABC genes in 151 patients who were diagnosed with ICP disease.

The present study has 4 major strengths. First, following the advent of WES technology, it has proven to be efficient in unearthing sequence variation across the MAF spectrum in obstetric and gynecological diseases [25, 37]. Using this method, we successfully identified novel candidate pathogenicity loci with known functional genes *ABCB4*, *ABCB11* [24] and *ABCC2*. In addition to these three genes, we also identified some novel variations in other genes in the ABC series. Our results revealed the genetic mutations of the first WES-identified ABC family genes associated with ICP disease. Second, to date, no studies have unraveled the genetic mutations in ABC genes of hepatic disease among pregnant women from a relatively large nationally representative sample (*n* = 151) in China. Using this same population, we previously identified a novel gene, *ANO8,* associated with ICP. Moreover, the clinical data of these patients are relatively complete, providing data support for association analysis between mutations and clinical data and subsequent functional verification. Finally, combining WES and clinical data favors deciphering the molecular mechanism of ICP disease.

Certainly, our study also has limitations. First, the WES approach needs a larger sample size to target low-frequency and rare variants. Otherwise, it may miss some valuable variants. Moreover, it also results in inaccurate MAFs for rare variants. However, the strict exclusion conditions guaranteed the selection of a defined cohort, such as employing the MAF in the databases and identifying 151 ICP cases and using 1029 local controls, combined with prediction tools. Second, the samples in this study were all derived from Jiangxi Province, which lacks geographic diversity and may limit the applicability of the generality of these results. However, this study is still valuable due to the high incidence (~ 5%) of ICP in Jiangxi. Next, the frequency of damaging mutations in 151 ICP samples was 10.60%, accounting for only a small portion of the genomic mutations of ICP disease. Therefore, we need to identify more genes/mutations associated with ICP. Finally, our results provided some possible interesting causative mutations; however, the causality between these potential pathogenic candidate loci and ICP disease needs to be verified by validation functional experiments.

Previous studies have detected genetic mutations in ICP primarily by Sanger sequencing or offspring studies based on a limited number of individuals. The rare (MAF < 0.01) variations that affect ICP have been more challenging to assess. Fortunately, WES emerged as an efficient approach to identify exonic mutations in targeted genes. Exonic variants, particularly missense, nonsense, and loss-of-function variants, tend to show the most dramatic effect sizes, possessing greater power for detection. In recent years, in obstetrics and gynecology, there have been a number of examples of using the WES method to search for key candidate genes and causative variants. For instance, Huusko et al., employing WES, revealed that *HSPA1L* is a genetic risk factor for spontaneous preterm birth [25]. To our knowledge, this study is the largest analysis to date of the role of mutations in the ABC series of genes in 151 ICP-susceptible patients.

Intriguingly, seven novel pathogenic loci in the *ABCB4*, *ABCB11* and *ABCC2* genes were simultaneously assigned to the damage group, suggesting the accuracy of our results. Several studies have shown that heterozygous missense mutations in *ABCB4*, especially low-frequency and rare variations, are commonly responsible for the occurrence of ICP disease, which is consistent with our results [38–40]. After filtering the frequency of the data, we identified a total of eight mutations with a MAF < 0.01, seven of which were heterozygous missense mutations and one of which was a nonsense mutation in *ABCB4*. The role of *ABCB11* in ICP has also been clearly identified, although its contribution seems less than that of *ABCB4*. A comprehensive analysis of multiple previous studies concluded that up to 5% of ICP cases harbor monoallelic mutations in *ABCB11* [41]. Our study confirmed the role of *ABCB11* and further expanded the role of the ABCB11 gene.

The function of *ABCC2* is related to ICP disease. Therefore, genetic variants of *ABCC2* may cause bile acid metabolism disorder. Our results demonstrated a small number of variations, in particular one novel pathogenic heterozygous (*ABCC2* Ser1342Tyr) variant. The study of Corpechot C et al. showed that genetic variants of *ABCC2* contribute to inherited cholestatic disorders [42]. Moreover, More et al. also found that the expression of *ABCC2* was more closely related to the livers from an alcohol cirrhosis cohort, indicating that *ABCC2* expression changed in liver-related disease [43], which is consistent with the fact that ICP is a liver disease. For the other three genes that were expressed differently, we found *ABCC6* to have a novel mutation, His1043Gln, which was located in the probably damaging group. Therefore, the *ABCC6* gene and the His1043Gln locus are of interest in the development of ICP disease. In our study, we failed to detect novel mutations in the *ABCE1* and *ABCG5* genes; therefore, we conjectured that this is probably because of the low frequency of novel mutations. It might also be that the known mutations in these two genes are associated with ICP, or other patterns of mutations lead to changes in the mRNA expression levels of the two genes. Moreover, compared to healthy samples, the levels of *ABCE1* and *ABCG5* mRNA expression were upregulated and downregulated in ICP, respectively. A reasonable explanation for this condition is that the unique functions of *ABCE1* and *ABCG5* lead to different mechanisms of ICP disease.

Because there have been relatively few studies on the genetic basis of ICP disease, many functional genes have not been mined. However, based on previous studies, several researchers found some evidence that certain genes may predispose patients to liver or ICP disease, such as *ABCB9* associated with hepatocellular carcinoma and *ABCG2* associated with ICP [44, 45]. Therefore, we hypothesized that genetic mutations in these unknown functional genes, excluding *ABCB4*, *ABCB11* and *ABCC2*, confer susceptibility to ICP, especially in the damaging group. These findings extended the knowledge on the role of mutations and strengthened the understanding of the genetic basis of ICP disease, including many additional ABC family genes.

Bacq et al. suggested that elevations in serum TBA, ALT and AST activity were identified among patients with ICP who have mutations in ABC transporter genes [46]. In agreement with this, our results confirmed that the mutation group containing 42 novel variants was associated with higher TBA, AST, DBIL, CHOL, TG and HDL, especially among the patients with ICP with 15 damaging novel mutations (Table 3, Fig. 7). Furthermore, Piatek K et al. suggested that the analysis of genotype coexistence pointed to the possibility of mutated variants of polymorphisms of genes having a summation effect on the development of ICP disease [47]. Consistent with this result, in our study, we found that six patients with ICP had two mutations at once. Notably, ICP harboring both mutations increased the average TBA level by 17.33, 20.22 and 50.32 μmol/L based on the difference in average TBA levels in ICP with one mutation, no mutation in ABC transporter genes and no mutation in healthy controls, respectively. As expected, AST, DBIL, CHOL, TG and HDL were also increased. A reasonable explanation for this situation is that double mutation has an additive effect on TBA levels, and this accumulation of TBA levels results in ICP exacerbation.

Moreover, significant differences between the wild type and mutant type were seen for the biochemical indexes, which suggested that the accumulation of TBA could lead to lipid abnormalities. Consistent with this result, researchers found that bile acids (BAs), which are well known for their amphipathic nature, are essential to lipid absorption and energy balance in humans [48–50]. Thus, taken together, our study provides additional support for the effect of mutations in ABC family genes on ICP disease and lipid metabolism.

Not surprisingly, to date, there are only a handful of reports of genetic analysis studies for ICP disease. The present findings not only enrich the molecular basis of the known functional genes (such as *ABCB4*, *ABCB11* and *ABCC2*) but also expand the new candidate genes (ABCA, ABCB, ABCC, and ABCD-ABCG) associated with ICP. Our results support the fact that mutations in ABC transporter genes lead to ICP disease. Therefore, further work on the genetic mutations involved in ICP pathogenesis has the potential to inspire novel therapies for patients with ICP.

Recently, there has been much interest in whether variations in bile salt concentrations and mutations in *ABCB4*/*ABCB11*/*ABCC2*/other new candidate genes could be biomarkers for various forms of drug-induced liver injury [51]. Furthermore, considering the negative effect of ABC mutations on maternal and fetal outcomes, it is important to genotype these mutations to genetically diagnose them in a timely manner and provide immediate attention and treatment for all ICP-susceptible people.

## Conclusion

To the best of our knowledge, this is the first study to conduct WES to reveal the genetic variants in ABC family genes associated with ICP disease. We detected 42 novel potential pathogenic mutations in 44 ABC family genes. Among them, seven loci were identified in *ABCB4*, *ABCB11* and *ABCC2*, and the remaining 35 loci were in other genes. In particular, the 15 novel mutations that were classified as the damaging group were the main focus of further study. Their functional validation and experimental verification need to be further investigated. Moreover, we detected genetic variants that were significantly associated with six biochemical indexes, including TBA, ALT, AST, DBIL, CHOL and TG (*P* < 0.05). Our findings provide new valuable insights into the genetic basis of ICP disease and suggest potential candidate variants for clinical diagnosis.

## Supplementary Information

**Additional file 1. **Correlation coefficients among twenty-six clinical features. The dots indicate the significant (*P* < 0.05) correlation coefficients between each pair of features. The size and colors separately represent the degree and direction of correlation coefficients.

## Data Availability

The datasets used and/or analyzed during the current study are available from the corresponding author on reasonable request.

## Abbreviations

ICP: Intrahepatic cholestasis of pregnancy
WES: Whole-exome sequencing
MAF: Minor allele frequency
TBA: Total bile acid
BWA: Burrows-Wheeler Aligner
GATK: Genome Analysis Toolkit
WBC: White blood cell
RBC: Red blood cell
PLT: Platelet
RDW.SD: Red blood cell distribution width SD
ALT: Alanine transaminase
AST: Aspartate transaminase
TBIL: Total bilirubin
DBIL: Direct bilirubin
IDBIL: Indirect bilirubin
CHOL: Total cholesterol
TG: Triglyceride
HDL: High-density lipoprotein
LDL: Low-density lipoprotein
