# Supplementary figures and images for: Whole-exome sequencing identifies novel mutations in ABC transporter genes associated with intrahepatic cholestasis of pregnancy disease: a case-control study
Source: BMC Pregnancy Childbirth. 2021 Feb 5;21:110. doi: 10.1186/s12884-021-03595-x (PMC7866704; doi:10.1186/s12884-021-03595-x)

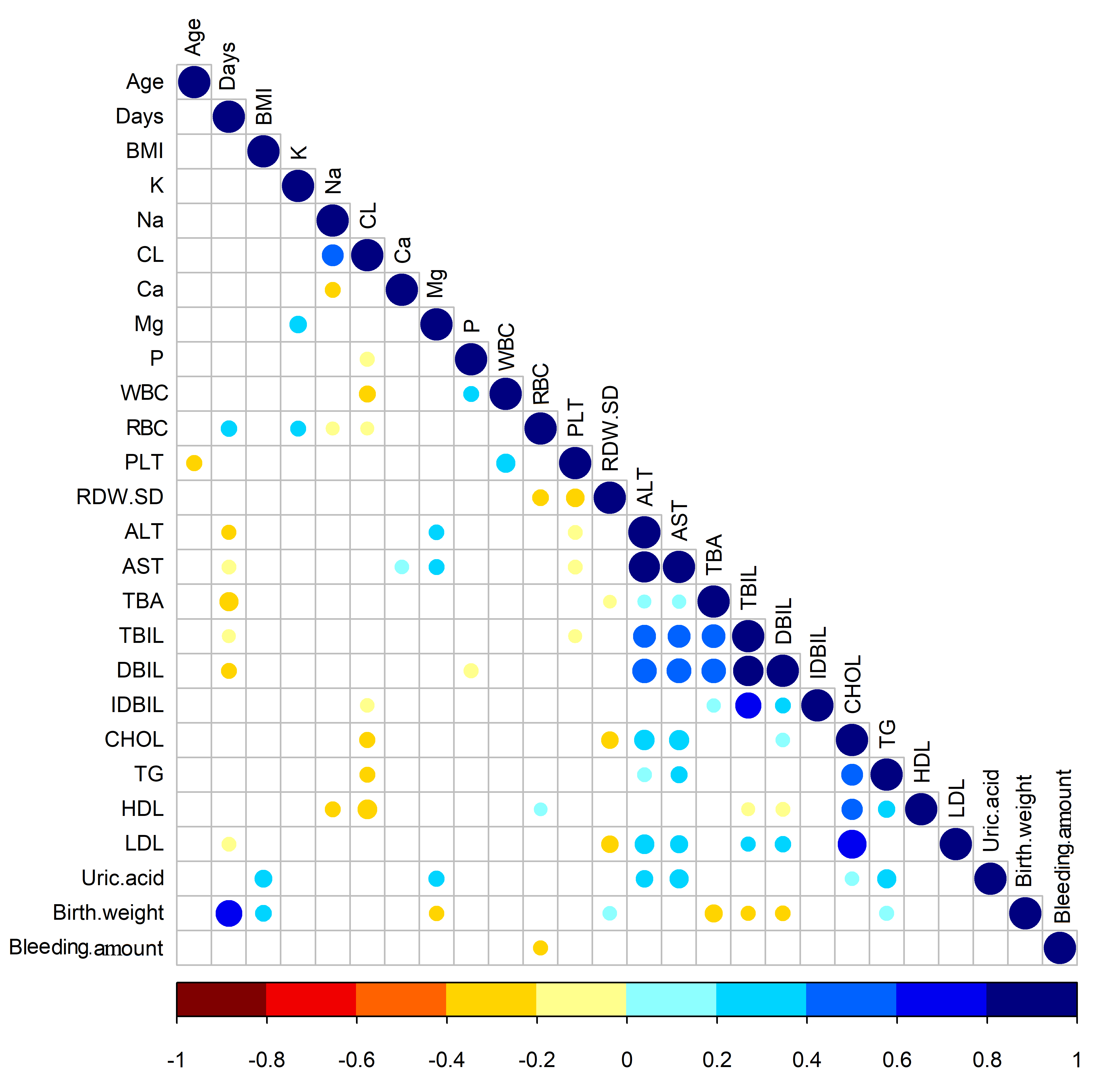

Supplement: Supplementary file 1 — Additional file 1. Correlation coefficients among twenty-six clinical features. The dots indicate the significant (P < 0.05) correlation coefficients between each pair of features. The size and colors separately represent the degree and direction of correlation coefficients. [file 12884_2021_3595_MOESM1_ESM.tif]
